# An H_2_O_2_ and MPO programmable responsive MRI probe for early detection of drug-induced acute kidney injury *via* spatiotemporal monitoring of renal oxidative stress and inflammation

**DOI:** 10.1016/j.redox.2026.104278

**Published:** 2026-06-25

**Authors:** Li He, Jia-Mi Li, Meng-Ting Li, Cai-Ju Zhang, Yu-Fan Lv, Jiao-Jiao Ma, Mao-Lin Zou, Bo Wu, Shi-Wen Huang, Gang Liu, Yong-Chang Wei, Dan Xu, Kai Deng

**Affiliations:** a Department of Radiology, Zhongnan Hospital of Wuhan University, Wuhan, 430071, China; b Department of Radiology, Renmin Hospital of Wuhan University, Wuhan, Hubei, 430060, China; c Department of Radiation and Medical Oncology, Zhongnan Hospital of Wuhan University, Wuhan, 430071, China; d Department of Nuclear Medicine, Zhongnan Hospital of Wuhan University, Wuhan University, Wuhan, 430071, China; e State Key Laboratory of Molecular Vaccinology and Molecular Diagnostics, State Key Laboratory of Vaccines for Infectious Diseases, Center for Molecular Imaging and Translational Medicine, Xiangan Biomedicine Laboratory, School of Public Health, Xiamen University, Xiamen, 361102, China; f Key Laboratory of Biomedical Polymers of Ministry of Education, Department of Chemistry, Wuhan University, Wuhan, 430072, China; g Department of Radiology, Hainan Affiliated Hospital of Hainan Medical University, Hainan, 570311, China

**Keywords:** Acute kidney injury, Spatiotemporal MRI, Early diagnosis, Oxidative stress, Inflammation

## Abstract

The sequential monitoring of two pathological processes has clinical significance for early detection of drug-induced acute kidney injury (AKI), but remains challenging due to the lack of spatiotemporal probes. Here, we developed a hydrogen peroxide (H_2_O_2_) and myeloperoxidase (MPO) programmable responsive magnetic resonance imaging (MRI) probe (PAH-Gd) for spatiotemporal monitoring of renal oxidative stress and inflammation. Upon exposure to H_2_O_2_, PAH-Gd transformed into a kidney-targeting moiety, H–Gd, which selectively accumulated in kidneys and moderately enhanced renal T1-weighted imaging (T1 WI) signal. Subsequently, the H–Gd could be oxidized by MPO to form dimers or adducts with nearby proteins, further enhancing renal T1 WI signal. We found that PAH-Gd could perform spatiotemporal monitoring of renal oxidative stress and inflammation at 12 h and 24 h post-Cisplatin (DDP) administration, enabling early detection of DDP-induced AKI at least 60 h earlier than that of standard clinical assays and permitting dynamic monitoring of renal injury progression. The spatiotemporal T1 WI overcame the limitations in detecting diverse pathological processes with a single MRI probe, accurately reporting the drug-induced AKI in the early stage and contributing to AKI prevention and treatment in the clinic.

## 1 Introduction

Acute kidney injury (AKI) is a clinical syndrome characterized by a rapid decline in kidney function caused by various etiologies, including nephrotoxin exposure. Notably, nephrotoxicity, such as cisplatin (DDP)-induced AKI, accounts for 20-30% of AKI clinical cases due to the widespread use of drugs [1]. Early detection of the initiation and progression of kidney damage is critical for guiding timely remedial measures to aid renal recovery and prevent severe complications, renal transplant, and even death [[2], [3], [4], [5]]. Currently, renal function in clinical practice is assessed by measuring serum creatinine (sCr) and blood urea nitrogen (BUN). However, these biomakers remain within the normal range until the glomerular filtration rate (GFR) has decreased by 50%, and thus only reflect renal dysfunction at a late stage of kidney injury [6,7]. Although renal biopsy can detect molecular changes associated with AKI at an early stage, the procedure is complex and carries potential risks, including hematuria, vascular embolism, and infection, which may lead to severe secondary damage, rendering it unsuitable for monitoring the kidneys of patients on routine medication. Therefore, given the inevitable use of certain or uncertain nephrotoxic drugs, the development of non-invasive methods for timely detecting the molecular changes of renal molecular information has important clinical significance for early diagnosis and recovery of drug-induced AKI.

To develop non-invasive imaging methods for early diagnosis, it is essential to comprehend the underlying mechanisms of drug-induced AKI. Oxidative stress, characterized by an excess of reactive oxygen species (ROS) that overwhelms antioxidant defenses, is a critical factor in triggering damage to cellular structure and function, and can even lead to cell death [8]. The oxidative stress within proximal tubular epithelial cells (PTECs) is widely recognized as the initiating insult and principal pathogenic driver of drug-induced acute kidney injury (AKI). In this context, the excess ROS, including hydrogen peroxide (H_2_O_2_), disrupts redox homeostasis, precipitating lipid peroxidation, protein and DNA oxidation, and mitochondrial dysfunction that culminate in tubular cells damage and death [[9], [10], [11]]. Accordingly, several molecular probes that image the ROS in PTECs have been used for early non-invasive diagnosis of drug-induced AKI [[12], [13], [14], [15], [16], [17]]. However, the sole detection of ROS in PTECs provides an incomplete picture of the initiation and progression of early AKI, which can easily lead to misdiagnosis [18]. Owing to the complex pathogenesis and rapid progression of drug-induced AKI, the oxidative stress in PTECs can lead to a series of cascading events, including apoptosis and necrosis of PTECs and inflammatory responses, thereby impairing kidney function through multiple pathways [[19], [20], [21]]. Additionally, ROS levels only reflect the extent of oxidative stress in specific regions of the kidney, such as renal tubules, making it impossible to directly assess the overall impact of nephrotoxic drugs on renal homeostasis. To overcome these limitations, identifying interconnected pathophysiological processes could lead to a more accurate and comprehensive assessment of the occurrence and development of kidney damage. Notably, renal inflammation, a systemic damaging response in the kidneys that can be triggered by oxidative stress, plays a crucial role in the initiation and progression of drug-induced AKI. The ROS-related by-products, including cytokines and chemokines, secreted by injured PTECs contribute to the inflammatory responses through recruiting and activating leukocytes, including macrophages and neutrophils, in renal tissues. These activated leukocytes produce large amounts of oxidative enzymes and substances, including myeloperoxidase (MPO), thus further exacerbating tubular cells and kidneys damage [22,23]. Furthermore, several studies have demonstrated that the inhibition of inflammatory stress can significantly alleviate drug-induced AKI. Thus, we hypothesized that developing a molecular probe capable of sequentially detecting the onset of oxidative stress and inflammatory responses in the kidneys would enable spatiotemporal monitoring of drug-induced AKI at an early stage.

Magnetic resonance imaging (MRI) with high resolution, deep penetration, and non-ionizing radiation is one of the most widely used modalities in clinical medicine for visually detecting disease by acquiring the signal of water protons in soft tissues [24]. However, current MRI technology can only depict the anatomical structures of late-stage renal damage in the clinic, even with the use of commercial gadolinium-based contrast agents, as it is unable to capture biological information at the cellular and molecular levels [25]. To improve the specificity and accuracy, smart contrast agents have been developed to specifically enhance the MRI signal in the kidneys for early detection of AKI based on the pathological biomarkers, including Fe^2+^ and ROS [26,27]. However, these probes predominantly target a single biomarker in the kidneys, limiting their ability to detect distinct pathological processes. Additionally, the “dual-locked” MRI contrast probes, which activate or enhance the MRI signal upon simultaneous stimulation with two biomarkers, offer an alternative approach but fail to distinguish diverse pathological processes that rely on reporting two independent biomarkers, respectively [[28], [29], [30]]. Therefore, the development of an MRI probe capable of sequentially detecting the onset of oxidative stress and inflammatory responses in the kidneys is required for spatiotemporal monitoring of drug-induced AKI in the early stage, although this remains a significant challenge.

**Fig. 1:**
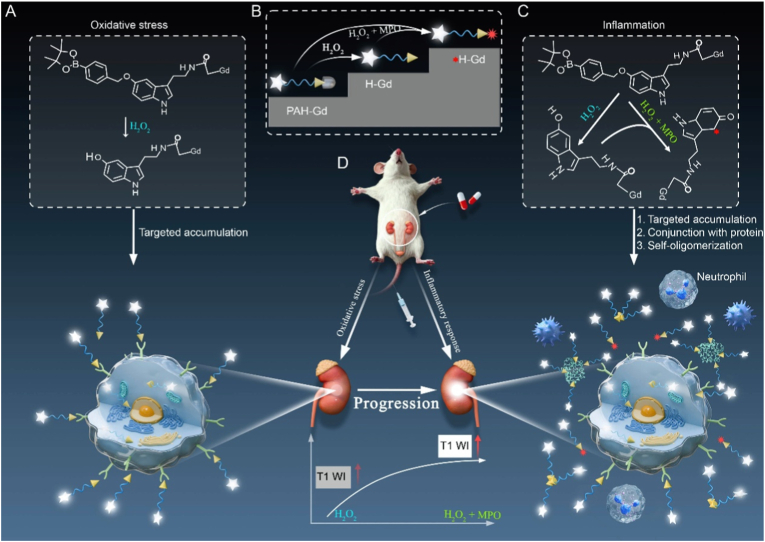
Schematic illustration of the PAH-Gd for early detection of DDP-induced AKI. (A) During oxidative stress, PAH-Gd undergoes oxidative decomposition in the presence of H_2_O_2_, forming 5-hydroxytryptamine-DOTA/Gd (H–Gd), which can be selectively taken up by renal tubular epithelial cells (PTECS). (B)The programmable response of PAH-Gd to H_2_O_2_ and MPO. The PAH-Gd is converted to H–Gd in the presence of H_2_O_2_, and further converted to H–Gd containing carbon-centered radicals in the presence of MPO plus H_2_O_2_. (C) During inflammation, PAH-Gd undergoes oxidative decomposition in the presence of H_2_O_2_ or forms H–Gd containing carbon-centered radicals in the presence of MPO plus H_2_O_2_. The H–Gd can be selectively taken up by renal tubular epithelial cells (PTECs). The H–Gd containing carbon-centered radicals can form dimers or generate adducts with nearby proteins. (D) The sequential detection of renal oxidative stress and inflammation with PAH-Gd for early detection of DDP-induced AKI. During oxidative stress, renal T1 WI is moderately enhanced by targeted accumulation of H–Gd in the kidneys. During inflammation, renal T1 WI ascended to higher levels through two pathways, including 1) the accumulation of Gd^3+^ in PTECs; 2) the formation of dimers or the generation of adducts with nearby proteins, which led to intensive retention and a slow tumbling effect of Gd^3+^ in kidneys.

As widely reported, we observed a cascade of renal oxidative stress and inflammatory response following cisplatin (DDP) administration, characterized by the sequential generation of H_2_O_2_ and MPO in the kidneys [[31], [32], [33]]. On this basis, we developed a hydrogen peroxide (H_2_O_2_) and myeloperoxidase (MPO) programmable responsive magnetic resonance imaging (MRI) probe (PAH-Gd) for early detection of drug-induced AKI *via* spatiotemporal monitoring of renal oxidative stress and inflammation, Fig. 1. The PAH-Gd was composed of four distinct components: 1,4,7,10-tetraazacyclododecane-N, N′, N″, N‴-tetraacetic acid (DOTA) group, gadolinium ions (Gd^3+^), 5-hydroxytryptamine (5-HT, MPO substrate), and phenylboronic acid pinacol ester (PAP, H_2_O_2_-responsive part). The 5-HT moiety enabled targeting of PTECs via 5-HT receptors on the cytomembrane; however, the targeting ability diminished when PAP blocked the active hydroxyl group of the 5-HT part. Under oxidative stress, PAH-Gd was oxidized by H_2_O_2_ to 5-hydroxytryptamine-DOTA/Gd (H–Gd), which promoted the accumulation of Gd^3+^ within PTECs *via* ligand-receptor interactions, thereby producing a moderate enhancement of renal T1 WI. Under inflammatory response, the renal T1WI increased to higher levels through a logical mechanism: 1) H_2_O_2_ oxidized PAH-Gd to H–Gd, leading to accumulation of Gd^3+^ in PTECs; 2) MPO further catalyzed oxidation of H–Gd in the presence of H_2_O_2_, forming dimers or generating adducts with nearby proteins, which led to intensive retention and slow tumbling effect of Gd^3+^ in kidneys. *In vivo*, we confirmed that PAH-Gd could accurately detect the occurrence and progression of AKI at an early stage by spatiotemporal monitoring of H_2_O_2_ and MPO in the kidneys at 12 h and 24 h after DDP administration. Notably, PAH-Gd exhibited rapid renal clearance with substantial excretion within 1 h after intravenous (*i.v.*) injection and induced negligible nephrotoxicity, indicating a favorable renal safety profile. Above all, PAH-Gd presented in this article provides strong imaging support for the non-invasive early diagnosis, prevention, and treatment of drug-induced AKI.

## 2 Results

### 2.1 Synthesis and characterization of PAH-Gd

The detailed synthetic route of PAH-Gd was depicted in Supplementary Fig. 1. Briefly, the hydroxyl group in 5-HT was first caged with H_2_O_2_-responsive part phenylboronic acid pinacol ester (PAP) to form PAH-BOC through Williamson ether synthesis. After tert-butyloxycarbonyl (BOC) deprotection, the PAH was further conjugated with DOTA-NHS to form PAH-DOTA. Lastly, the PAH-DOTA chelated with Gd^3+^ to form PAH-Gd. The gadolinium 1,4,7,10-tetraazacyclododecane-1,4,7,10-tetraacetate (Gd-DOTA), a Gd^3+^ complex with high thermodynamic stability and kinetic inertness, was used as the core of PAH-Gd. 5-hydroxytryptamide (5-HT) was exploited as the targeting moiety for PTECs and the substrate moiety for MPO. For one thing, 5-HT, a neurotransmitter primarily found in the gastrointestinal tract and central nervous system, has recently been identified to regulate renal metabolism and homeostasis through 5-HT receptors [[34], [35], [36]]. For another, 5-HT, an efficient substrate for MPO, could form aggregates through dimerization or form adducts via attaching to nearby proteins when oxidized in the presence of MPO + H_2_O_2_ [[37], [38], [39]]. The 5-HT conjugated with DOTA-NHS and then chelated with Gd^3+^ to form H–Gd as a control probe (Supplementary Fig. 2). The successful preparation of the intermediates and final products was confirmed through ^1^H Nuclear magnetic resonance (^1^H NMR) and mass spectrometry (MS) (Supplementary Figs. 3–9).

**Fig. 2:**
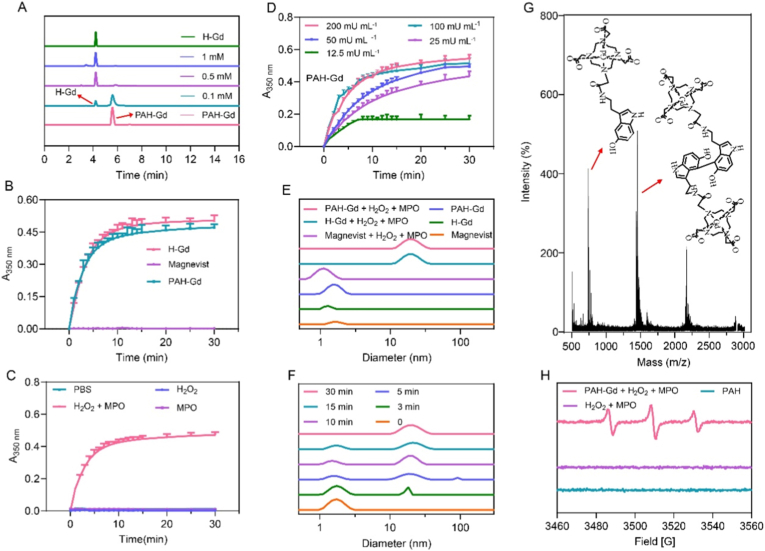
The programmable response of PAH-Gd to H_2_O_2_ and MPO + H_2_O_2_. (A) HPLC analysis of the PAH-Gd treated with different concentrations of H_2_O_2_ (0.1, 0.5, 1 mM). (B) The UV–Vis absorbance at 350 nm for PAH-Gd, H–Gd and Magnevist in the presence of MPO (200 mU mL^−1^) + H_2_O_2_ (1 mM) (0.1 M Tris buffer, pH = 7.4, 37 °C). (C) The UV–Vis absorbance at 350 nm for PAH-Gd in the presence of MPO (200 mU mL^−1^) + H_2_O_2_ (1 mM), H_2_O_2_ (1 mM), and MPO (200 mU mL^−1^) (0.1 M Tris buffer, pH = 7.4, 37 °C). (D) The UV–Vis absorbance at 350 nm for PAH-Gd in the presence of MPO + H_2_O_2_ (1 mM) with different concentrations of MPO (0.1 M Tris buffer, pH = 7.4, 37 °C). (E) The size distribution of PAH-Gd, H–Gd, and Magnevist in the presence of MPO + H_2_O_2_ (0.1 M Tris buffer, pH = 7.4, 37 °C). (F) The size distribution of PAH-Gd after incubation with MPO (200 mU mL^−1^) + H_2_O_2_ (1 mM) (0.1 M Tris buffer, pH = 7.4, 37 °C) for different times. (G) The MALDI-TOF spectra of PAH-Gd under stimulation with MPO (200 mU mL^−1^) + H_2_O_2_ (1 mM) (0.1 M Tris buffer, pH = 7.4, 37 °C). (H) The EPR spectra of the PAH-Gd with or without MPO (200 mU mL^−1^) + H_2_O_2_ (1 mM) (0.1 M Tris buffer, pH = 7.4, 37 °C) stimulation. Data are means ± SD (n = 3).

As previously reported, PAP was highly sensitive to H_2_O_2_, undergoing oxidation and exposing the caged group when exposed to H_2_O_2_. To verify this report, HPLC analyzed the reaction mixture of PAH-Gd and H_2_O_2_, Fig. 2A and Supplementary Fig. S10. In the presence of H_2_O_2_ (0.1 mmol), the HPLC peak of PAH-Gd at 5.6 min decreased, while a new peak at 4.2 min corresponding to the formation of H–Gd increased. After incubation with 1 mM H_2_O_2_ for 1 min, the HPLC peak of PAH-Gd at 5.6 min disappeared, accompanied by the H–Gd peak reaching its maximum at 4.2 min, indicating that PAH-Gd was highly sensitive to H_2_O_2_. To investigate the response of PAH-Gd under physiological concentrations of H_2_O_2_, we used HLPC to analyze the structural changes of PAH-Gd after co-incubation with 0.01, 0.05, and 0.1 mM H_2_O_2_ for 10 min (Supplementary Fig. 11). We observed that PAH-Gd remained stable at H_2_O_2_ concentrations 500 times (0.01 mM) higher than physiological levels (0.05-0.7 μM). These results revealed that PAH-Gd was stable under normal physiological conditions. To further validate the oxidation behavior of PAH with H_2_O_2_ and MPO + H_2_O_2_, the UV−vis spectroscopy was employed to monitor changes in absorbance at 350 nm, Fig. 2B–D. The 5-HT in monomer state showed no absorbance at 350 nm, whereas in its aggregated state, it showed strong UV–Vis absorbance at 350 nm due to dimers extending the π-conjugation of the indole system. Under MPO (200 mU mL^−1^) + H_2_O_2_ (1 mM) stimulation, the absorbance at 350 nm sharply increased and reached a plateau at 15 min for H–Gd and PAH-Gd, validating the formation of dimers. Meanwhile, the formation of dimers was positively correlated with the concentration of MPO and H_2_O_2_ (Fig. 2D and Supplementary Fig. 12). While negligible UV–Vis absorbance at 350 nm was observed in the mixture of Magnevist and MPO (200 mU mL^−1^) + H_2_O_2_ (1 mM). Although PAH-Gd was sensitive to H_2_O_2_, there was no UV–Vis absorbance at 350 nm for the mixture of PAH-Gd and H_2_O_2_ (1 mM), demonstrating the high stability of H–Gd in the presence of H_2_O_2_. Additionally, the single MPO also did not influence the stability of PAH-Gd. The formation of dimers for PAH-Gd in the presence of MPO + H_2_O_2_ was further investigated with dynamic light scattering (DLS), Fig. 2E–F. The size of PAH-Gd increased from 1.3 nm to 15.5 nm under MPO (200 mU mL^−1^) + H_2_O_2_ (1 mM) stimulation, which was consistent with the mixture of H–Gd and MPO (200 mU mL^−1^) + H_2_O_2_ (1 mM). However, the single H_2_O_2_ (1 mM) did not influence the size of PAH-Gd and H–Gd. The quantitative analysis using MALDI-TOF revealed that MPO + H_2_O_2_ could induce the oxidation and polymerization of PAH-Gd, resulting in the formation of dimers, Fig. 2G. The dimerization of the indole system in PAH-Gd was ascribed to the formation of radicals in the presence of H_2_O_2_ and MPO. To confirm the generation of radicals for PAH-Gd in the presence of H_2_O_2_ and MPO, the 5,5-dimethyl-1-pyrroline N-oxide (DMPO) was used as a trapping agent in electron paramagnetic resonance (EPR) analysis. DMPO can efficiently capture radicals to yield DMPO^−•^, and displays a typical ESR signal of 1: 1: 1 peak or a 1: 2: 2: 1 peak under a static magnetic field. In the presence of H_2_O_2_ and MPO, a strong triplet peak of TEMPO was observed for PAH-Gd, revealing the generation of radicals, Fig. 2H. Collectively, these results revealed that PAH-Gd exhibited programmable responses to H_2_O_2_ and MPO + H_2_O_2_.

### 2.2 *In vitro* imaging performance of PAH-Gd

**Fig. 3:**
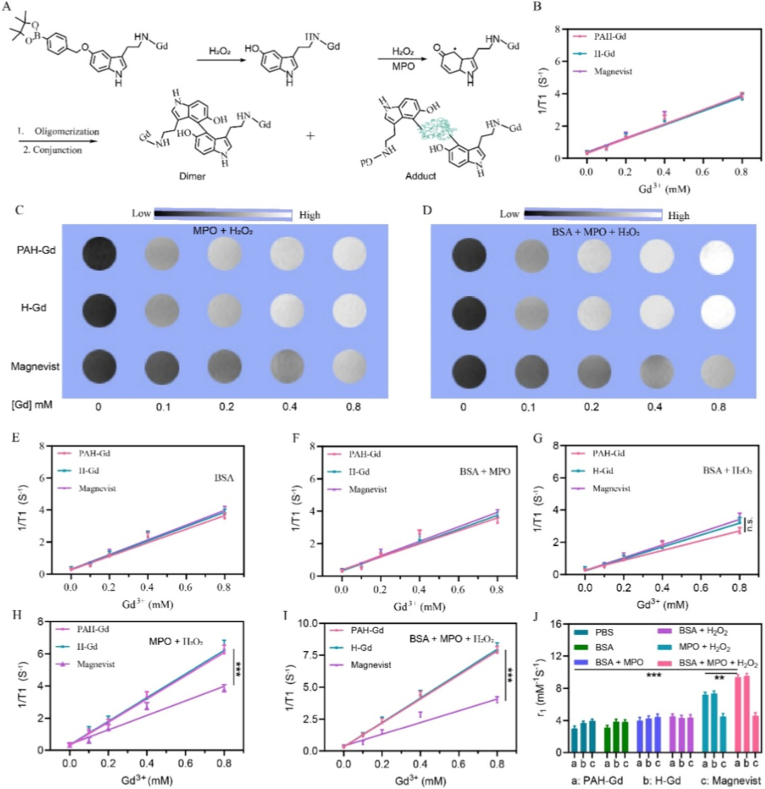
The *in vitro* performance of PAH-Gd on T1 WI. (A) Schematic illustration of the chemical reaction process of PAH-Gd under stimulation with H_2_O_2_ and MPO + H_2_O_2_; (B) The longitudinal relaxation rates R1 (1/T1) of PAH-Gd, H–Gd and magnevist at different concentrations of Gd^3+^. The T1 WI phantom images of PAH-Gd, H–Gd and magnevist in the absence (C) and presence of BSA (D), following MPO + H_2_O_2_ stimulation. (E) The longitudinal relaxation rates R1 (1/T1) of PAH-Gd, H–Gd and magnevist in the presence of BSA. The longitudinal relaxation rates R1 (1/T1) of PAH-Gd, H–Gd and magnevist in the presence of BSA, following MPO (F) and H_2_O_2_ (G) stimulation. The longitudinal relaxation rates R1 (1/T1) of PAH-Gd, H–Gd and magnevist in the absence (H) and presence (I) of BSA, following MPO + H_2_O_2_ stimulation. (J) The T1 relaxivity (r1) of PAH-Gd, H–Gd and magnevist after different treatments. Data are means ± SD (n = 3). ∗∗∗*P* < 0.001; n.s., not significant.

The longitudinal relaxation (T1) relaxivity of Gd-based molecular MRI probes can be dominated by their concentration, degree of polymerization, and extent of protein binding, which modulate molecular tumbling, rotational correlation time, and water-exchange dynamics. Considering PAH-Gd under simultaneous stimulation with MPO + H_2_O_2_ could generate dimers or form adducts with adjacent proteins (Fig. 3A), we anticipated that this process could slow the tumbling effect of Gd^3+^ and reinforce the T1 relaxivity. To investigate whether the formation of dimers and adducts could improve T1 relaxivity, the T1 WI phantom was conducted on a 5.0 T scanner at room temperature. T1 WI is a magnetic resonance sequence that differentiates tissue types by exploiting differences in longitudinal relaxation times. It primarily reflects the water and fat content of tissues, as well as specific signal changes in certain lesions. The T1 WI phantom images of PAH-Gd and Magnevist at different concentration of Gd^3+^ (0, 0.1, 0.2, 0.4, 0.8 mM) were firstly obtained, Fig. 3B and Supplementary Fig. 13A). Consistent with other Gd-based molecular probes, the longitudinal relaxation rates R1 (1/T1) of PAH-Gd and H–Gd were consistent with that of Magnevist, showing Gd concentration ([Gd]) dependent, and linearly reinforced with increasing Gd concentration, Fig. 3B. Subsequently, the T1 WI phantom images of PAH-Gd in the presence of H_2_O_2_ (1 mM) and MPO (200 mU mL^−1^) were obtained (Fig. 3C–D and Supplementary Fig. 13B–D). It was obvious that the co-treatment with MPO + H_2_O_2_ increased the R1 of PAH-Gd and H–Gd across all concentrations of Gd^3+^, Fig. 3E–I. In the presence of MPO + H_2_O_2_, the higher R1 of PAH-Gd and H–Gd was observed by the addition of BSA, ascribing to the slow tumbling of Gd^3+^ in dimers and adducts. However, the treatment with MPO + H_2_O_2_ showed a negligible change in the R1 of Magnevist at any concentration of Gd^3+^, regardless of the presence or absence of BSA. The longitudinal molar relaxivity (r_1_) was further calculated from the data in Fig. 3E–I according to the equation r_1_ = Δ*R*1/Δ[Gd^3+^], where the longitudinal relaxation rate R1 = 1/T1. In the presence of MPO + H_2_O_2_, the r_1_ of PAH-Gd (7.21 ± 0.30 mM^−1^ s^−1^) and H–Gd (7.33 ± 0.34 mM^−1^ s^−1^) showed a slight increase to 1.60-fold and 1.62-fold higher than that of Magnevsit (4.51 ± 0.37 mM^−1^ s^−1^), respectively. Significantly, the BSA further augmented the r1 relaxivity of PAH-Gd (9.37 ± 0.32 mM^−1^ s^−1^) and H–Gd (9.53 ± 0.33 mM^−1^ s^−1^) when MPO and H_2_O_2_ were both present. Conversely, BSA had no effect on the r1 relaxivity of PAH-Gd (3.99 ± 0.39 mM^−1^ s^−1^ or 4.53 ± 0.31 mM^−1^ s^−1^) or H–Gd (4.28 ± 0.32 mM^−1^ s^−1^ or 4.33 ± 0.33 mM^−1^ s^−1^) in the sole presence of either H_2_O_2_ or MPO. Additionally, in the absence of MPO + H_2_O_2_, BSA has no effect on the r_1_ of PAH-Gd, indicating no significant binding occurred between BSA and PAH-Gd. Collectively, the PAH-Gd showed high potential for detecting MPO by enhancing T1 WI in the presence of H_2_O_2_.

**Fig. 4:**
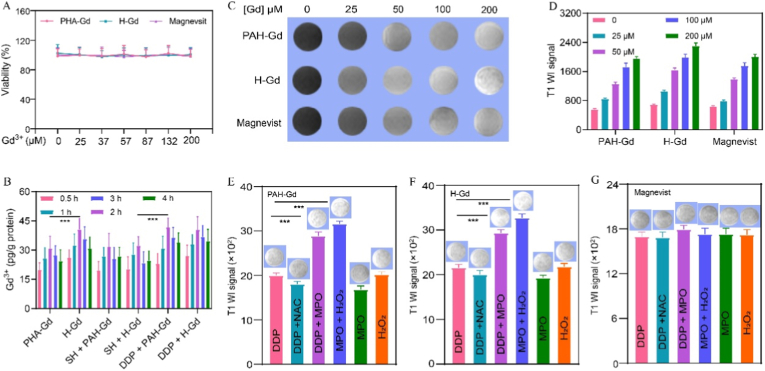
The cellular performance of PAH-Gd on T1 WI. (A) The cytotoxicity of PAH-Gd, H–Gd and Magnevist on HK-2 cells. (B) The ICP analysis of cellular concentration of Gd^3+^ after incubation with PAH-Gd, H–Gd and Magnevist for different times. The T1 WI phantom images (C) and corresponding T1 WI signal (D) of HK-2 cells treated with PAH-Gd, H–Gd and Magnevist for 2 h. (E-G) The T1 WI phantom images and corresponding T1 WI signal of HK-2 cells after different treatments. Data are means ± SD (n = 3). ∗∗∗*P* < 0.001.

The low cytotoxicity is imperative for probes *in vitro* and *in vivo*. To measure the cytotoxicity of PAH-Gd, the MTT assay was applied to detect the viability of HK-2 cells after incubation with PAH-Gd. The MTT results revealed that negligible cytotoxicity of PAH-Gd on HK-2 cells even at the concentration of Gd^3+^ up to 200 μM (98.36 ± 10.51%), Fig. 4A. Before evaluating the PAH-Gd as an MRI probe to detect H_2_O_2_ and MPO + H_2_O_2_ *in vitro*, the cellular uptake of PAH-Gd was first measured with the inductively coupled plasma atomic emission spectrometry (ICP-AES) at different incubation times, Fig. 4B. At 2 h, the concentration of Gd^3+^ in PAH-Gd treated HK-2 cells was 1.3-fold lower than that in H–Gd treated HK-2 cells. Upon pretreatment with the 5-HT receptor antagonist sarpogrelate hydrochloride (SH) (20 μM) for 30 min, the concentration of Gd^3+^ in H–Gd treated HK-2 cells was consistent with that in PAH-Gd treated HK-2 cells, revealing that the PAP could diminish the receptor-ligand interactions between H–Gd and 5-HT receptor [39,40]. Furthermore, the ICP results revealed that upon pretreatment with DDP (10 μM) for 12 h, the concentration of Gd^3+^ in PAH-Gd treated HK-2 cells was consistent with that in H–Gd treated HK-2 cells, which was attributed to the oxidation of PAH-Gd to H–Gd in the presence of H_2_O_2_.

The performance of PAH-Gd in detecting H_2_O_2_ and MPO *in vitro* was determined using a 5.0 T scanner at room temperature, Fig. 4C–G. The T1 WI signal of HK-2 cells treated with PAH-Gd was positively correlated with the concentration of Gd^3+^. The T1 WI signal in HK-2 cells treated with PAH-Gd was weaker than that of HK-2 cells treated with H–Gd due to the lower intracellular Gd^3+^ concentration. However, upon pretreatment with DDP (10 μM), the T 1WI signal in HK-2 cells treated with PAH-Gd (2000.78 ± 53.90) was close to that of HK-2 cells treated with H–Gd (2157.92 ± 72.3). However, the addition of antioxidant N-Acetyl-l-cysteine (NAC) decreased the T1 WI signal in HK-2 cells treated with DDP and PAH-Gd (1800.73 ± 63.30), ascribing to the scavenging of H_2_O_2_. Furthermore, the results revealed that the pretreatment of MPO (100 mU mL^−1^) and DDP (10 μM) further reinforced T1 WI signal in HK-2 cells treated with PAH-Gd (2892.43 ± 87.30) and H–Gd (3273.58 ± 91.20). This amplification represented a 1.6-fold and 1.8-fold increase, respectively, compared to HK-2 cells treated with Magnevist. Meanwhile, PAH-Gd exhibited a comparable T1 WI signal in HK-2 cells pretreated with MPO + DDP and MPO + H_2_O_2_. However, the single pretreatment with MPO showed negligible changes in the T1 WI signal of HK-2 cells treated with H–Gd, a finding consistent with observations in HK-2 cells treated with DDP and PAH-Gd. ICP-AES results revealed the concentration of Gd^3+^ in HK-2 cells separately treated with MPO + DDP + PAH-Gd (42.36 ± 4.32 pg/g protein), MPO + DDP + H–Gd (43.74 ± 6.52 pg/g protein), MPO + H_2_O_2_ + PAH-Gd (41.63 ± 5.80 pg/g protein), MPO + H_2_O_2_ + H–Gd (44.52 ± 5.40 pg/g protein) was nearly to that in HK-2 cells treated with H–Gd (40.56 ± 5.69 pg/g protein) (Supplementary Fig. 14), which suggested that the enhanced T1 WI signal of PAH-Gd in HK-2 cells treated with MPO + DDP and MPO + H_2_O_2_ was not only ascribed to the increase of Gd^3+^ concentration *via* the receptor-ligand interactions, but also might benefit from the formation of dimers and adducts. Together, the PAH-Gd exhibited excellent performance in distinguishing H_2_O_2_ and MPO + H_2_O_2_ by mediating sequential T1 WI *in vitro*.

### 2.3 DDP-induced sequential upregulation of H_2_O_2_ and MPO in kidneys

**Fig. 5:**
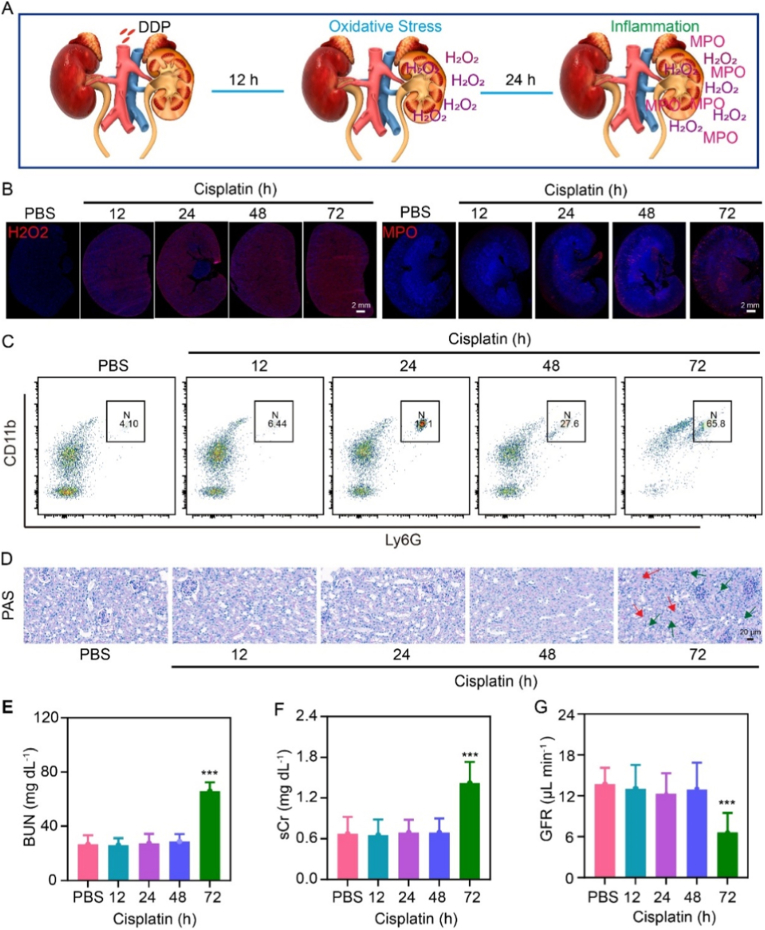
The mechanism of DDP-induced AKI. (A) Schematic illustration of the sequential upregulation of renal H_2_O_2_ and MPO in DDP (20 mg/kg)-treated mice. (B) Immunofluorescence staining of kidneys for H_2_O_2_ and MPO detection of mice after DDP-treatment for different times. (C) The FCM analysis of neutrophils in kidneys of mice after DDP-treatment for different times. (D) The periodic acid-schiff (PAS) staining of kidneys in mice treated with DDP (20 mg/kg) for different times. The casts and damaged tubules were marked with red arrows and green arrows, respectively. The levels of BUN (E) and sCr (F) in the serum of mice treated with DDP for different times. (G) The glomerular filtration rate (GFR) of mice treated with DDP (20 mg/kg) for different times. Data are means ± SD (n = 3). ∗∗∗*P* < 0.001.

In drug-induced AKI, oxidative stress and inflammation are key contributors [41]. H_2_O_2_ serves as a central early oxidant and signaling mediator, promoting tubular cell death via mitochondrial/endoplasmic reticulum stress-related apoptosis, necrosis, and ferroptosis. Additionally, H_2_O_2_ triggers inflammatory mediators (e.g., TNF-α, IL-6) that recruit neutrophils and monocytes, amplifying oxidative stress and tissue damage (Fig. 5A). The immunofluorescent staining of renal tissues revealed that the renal H_2_O_2_ red fluorescence was upregulated at 12 h post DDP (20 mg kg^−1^) administration, while negligible MPO red fluorescence was observed in kidneys, demonstrating renal H_2_O_2_ generation preceded MPO following cisplatin treatment, Fig. 5B. Subsequently, the renal MPO, secreted by the activated neutrophils, red fluorescence was observed at 24 h post DDP administration. Although the renal H_2_O_2_ red fluorescence intensity at 24 h was stronger than that at 12 h, it showed small changes at 48 and 72 h. Conversely, the renal MPO red fluorescence intensity at 48 and 72 h was further enhanced, revealing the persistent inflammatory response. Consistently, the FCM results revealed that the renal infiltration of neutrophils was upregulated at 24 h post DDP administration, Fig. 5C. Therefore, the single detection of renal H_2_O_2_ was difficult to evaluate the progression of DDP-induced AKI. The renal PAS staining revealed that the apparent damage to the tubules was observed until 72 h post-DDP administration, Fig. 5D. The renal function was further analyzed by clinical methods, including serum creatinine (sCr) and blood urea nitrogen (BUN) in blood. The sCr and BUN in DDP-treated mice had negligible change in comparison with PBS-treated mice at 12, 24, and 48 h. However, the sCr and BUN in mice treated with DDP for 72 h exhibited a statistically significant increase, which was 2.1-fold and 2.4-fold higher than that in mice treated with PBS, Fig. 5E–F. The upregulation of the sCr and BUN in blood revealed a nearly 50% decline in renal function, Fig. 5G. Collectively, these results demonstrated that the sequential upregulation of H_2_O_2_ and MPO in kidneys has the potential to guide early detection of the onset and progression of DDP-induced AKI.

### 2.4 *In vivo* imaging performance of PAH-Gd

**Fig. 6:**
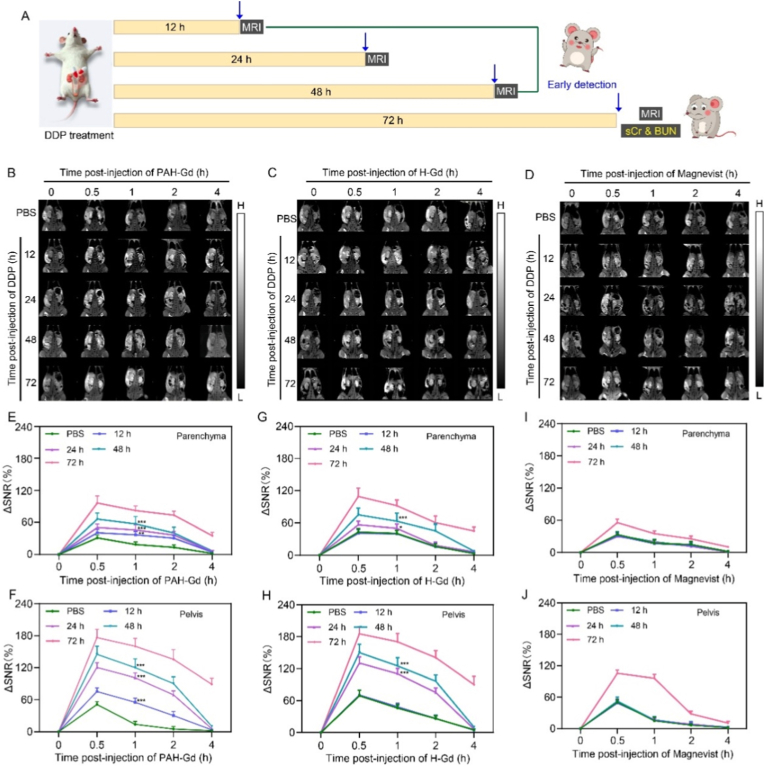
The early detection of the occurrence and progression of DDP-induced AKI *in vivo*. (A) Schematic illustration of *in vivo* protocol. The representative T1 WI phantom images of mice treated with or without DDP (20 mg/kg) for 12, 24, 48, and 72 h, following *i.v.* injection of PAH-Gd (B), H–Gd (C) and Magnevist (D). The ΔSNR (%) of parenchyma and pelvis calculated from T1 WI phantom images of mice treated with or without DDP(20 mg/kg) for 12, 24, 48, and 72 h, following *i.v.* injection of PAH-Gd (E-F), H–Gd (G-H), and Magnevist (I-J). Data are means ± SD (n = 3). ∗*P* < 0.05, ∗∗∗*P* < 0.001.

To study the performance of PAH-Gd in detecting DDP-induced AKI, DDP was intraperitoneally injected into living mice at a nephrotoxic dosage (20 mg kg^−1^), followed by *i.v.* injection of PAH-Gd, H–Gd, and Magnevist, respectively, at different time points (12, 24, 48, and 72 h) post-administration with DDP, Fig. 6A. To investigate the imaging performance of PAH-Gd in the diagnosis of DDP-induced AKI, the renal clearance of PAH-Gd, H–Gd, and Magnevist was first measured. The coronal T1 WI of the healthy mice was acquired after intravenous (*i.v.*) injection of PAH-Gd, H–Gd, and Magnevist (Gd^3+^ = 50 μmol kg^−1^), respectively, Fig. 6B–D. The representative T1 WI images exhibited strong contrast enhancement in the kidneys, revealing that PAH-Gd, H–Gd, and Magnevist were mainly excreted via the kidneys after *i.v.* injection. Additionally, the PAH-Gd and Magnevist showed a transient and weak T1 bright enhancement in parenchyma (cortex plus medulla) and pelvis regions of kidneys, while H–Gd exhibited evident T1 bright enhancement in parenchyma and pelvis for 2 h. The results revealed that PAH-Gd and Magnevist were rapidly eliminated from the kidneys to the bladder after *i.v.* injection, while H–Gd exhibited long retention in the kidneys after *i.v.* injection due to the high expression of the 5-HT receptor in the kidneys. After *i.v.* injection of PAH-Gd for 3 days, the HE staining of major organs exhibited negligible cell death or tissue damage, validating the high biocompatibility of PAH-Gd (Supplementary Fig. 15).

In the AKI mice model, the H–Gd induced T1 enhancement in the renal parenchyma and pelvis of mice post-treatment with DDP for 12 h was similar to that of mice treated with PBS. The renal T1 bright enhancement for H–Gd was ascribed to the expression of 5-HT receptors in the kidneys. Therefore, H–Gd failed to distinguish the upregulation of renal H_2_O_2_ in mice at 12 h post-treatment with DDP. In contrast, PAH-Gd induced a significant T1 bright signal enhancement in the renal parenchyma and pelvis of mice treated with DDP for 12 h, compared to PBS-treated mice. This enhancement is attributed to the oxidation of PAP in PAH-Gd, which restored the receptor-ligand binding interaction. Additionally, the T1 WI enhancement for PAH-Gd was comparable to that of the control probe H–Gd after DDP treatment for 12 h. These results revealed that PAH-Gd was capable of accurately detecting the early generation of H_2_O_2_ in the kidneys of mice treated with DDP for 12 h. With prolonged DDP administration, PAH-Gd further reinforced the T1 bright signal in renal parenchyma and pelvis, whereas Magnevist showed negligible enhancement before DDP-treatment for 72 h. At 72 h post-DDP administration, the reinforced T1 bright signal for Magnevist was ascribed to a decrease in renal function, which slowed the renal excretion of Magnevist. Furthermore, the △SNR (change in signal-to-noise ratio, %) in renal parenchyma and pelvis was calculated from T1 WI images for semiquantitative analysis. After *i.v.* injection of PAH-Gd for 1 h, the △SNR in renal parenchyma (36.8 ± 6.53%) and pelvis (55.2 ± 7.83%) of mice treated with DDP for 12 h was 1.9-fold and 4-fold higher than that of mice treated with PBS, Fig. 6E–J. After 24 h post-treatment with DDP, the △SNR for PAH-Gd in renal parenchyma and pelvis was further enhanced to 45.9 ± 9.12% and 101.2 ± 8.45%. The further reinforced renal △SNR for PAH-Gd was attributed to the elevation of H_2_O_2_ and MPO in the kidneys (Fig. 5B), which promoted the recovery of the receptor-ligand interaction between PAH-Gd and 5-HT receptor, and induced H–Gd to form dimers or adducts. Together, the results revealed that PAH-Gd could detect the initiation of oxidative stress and inflammation in the kidneys *via* stepwise enhancement of T1 WI.

**Fig. 7:**
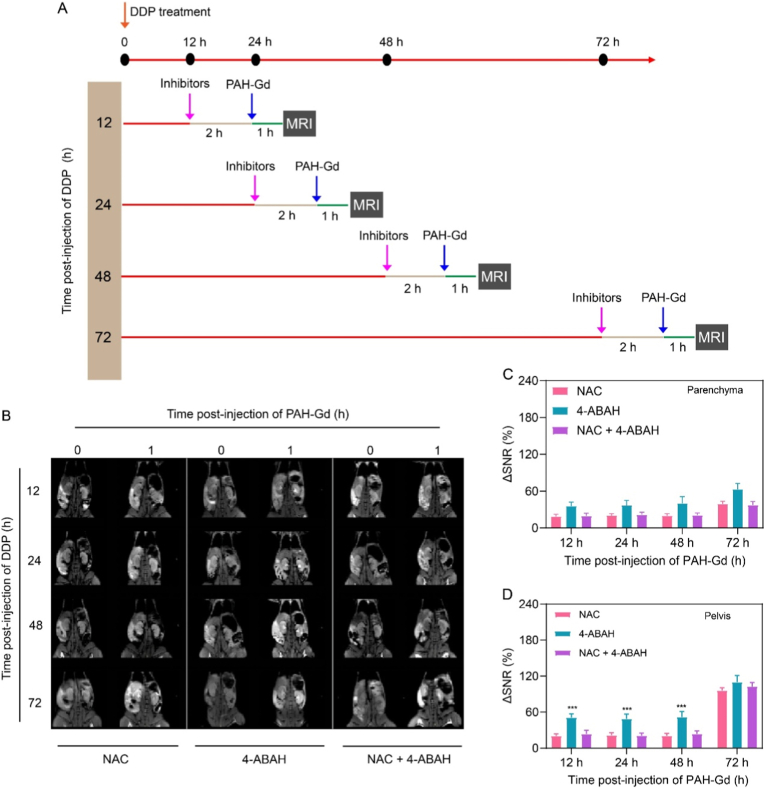
The influence of the inhibitor on renal T1 WI *in vivo*. (A) Schematic illustration of *in vivo* TI WI in the presence of inhibitors, including NAC, 4-ABAH, and NAC + 4-ABAH. (B) The T1 WI phantom images of mice treated without or with DDP (20 mg kg^−1^) for different times, following *i.v.* injection of PAH-Gd for 1 h. The ΔSNR (%) of parenchyma (C) and pelvis (D) calculated from T1 WI phantom images of mice treated with or without DDP(20 mg/kg) for 12, 24, 48, and 72 h, following *i.v.* injection of PAH-Gd for 1 h. Data are means ± SD (n = 3). ∗∗∗*P* < 0.001.

To further validate that PAH-Gd could spatiotemporally detect renal oxidative stress and inflammation, the NAC (antioxidant) and 4-ABAH (MPO inhibitor) were injected separately or together into mice 2 h before acquiring T1 WI, Fig. 7A. In the presence of NAC, the △SNR for PAH-Gd in renal parenchyma and pelvis of mice treated with DDP for 12, 24 and 48 h was consistent with that of normal mice, Fig. 7B–D. Meanwhile, the NAC and NAC + 4-ABAH had similar influence on the renal △SNR for PAH-Gd of mice treated with DDP, demonstrating the programmable response of PAH-Gd to H_2_O_2_ and MPO + H_2_O_2_. Although 4-ABAH did not affect the renal △SNR for PAH-Gd in mice treated with DDP for 12 h, it reduced the renal △SNR for PAH-Gd in mice treated with DDP for 24 and 48 h to levels comparable to those in mice treated with DDP for 12 h, due to inhibition of renal MPO activity. It revealed that PAH-Gd was highly sensitive to renal H_2_O_2_. Furthermore, the heightened renal △SNR for PAH-Gd in mice treated with DDP for 24 and 48 h was attributed to the increased expression of MPO in the kidneys, even though the continuous elevation of renal H_2_O_2_ at these same time points, Figs. 5B and 6E–F. These results further revealed that PAH-Gd mediated spatiotemporal T1 WI was positively correlated with the sequential production of H_2_O_2_ and MPO in kidneys. Collectively, PAH-Gd could early predict the occurrence and progression of DDP-induced AKI by sequentially detecting the initiation of two pathologic mechanisms with spatiotemporal T1 WI.

## 3 Conclusion

Nephrotoxicity accounts for 20-30% of AKI clinical cases that can progress to severe complications, renal transplant, and even fatality through multiple interlinked pathological processes. The noninvasive diagnosis of drug-induced AKI in the early stage is crucial for renal treatment and recovery, and it depends on comprehending the underlying mechanism of drug-induced AKI. During the early stages of DDP (20 mg/kg) administration, H_2_O_2_ levels in mouse kidney tissues increased significantly, indicating that H_2_O_2_ generation is the initiating factor in DDP-induced kidney injury. However, as time progressed, the changes in renal H_2_O_2_ levels became less pronounced, while kidney tissue damage continued to worsen. This suggests that H_2_O_2_ detection alone is insufficient for accurately assessing the progression of drug-induced AKI [[29], [30], [31]]. Further analysis revealed that after DDP administration for 24 h, the kidney exhibited extensive inflammatory cell infiltration and significant upregulation of MPO. These findings indicated that MPO, as a marker of inflammatory cell activation, could serve as a reliable indicator for evaluating the progression of kidney damage, thereby supplementing the limitations of H_2_O_2_ detection. Thus, the spatiotemporal detection of the initiation of oxidative stress and inflammatory response in the kidneys might contribute to monitoring and preventing the occurrence and development of drug-induced AKI in the early stage.

Noninvasive monitoring of single biomarkers, such as Fe^2+^ or ROS, in the kidneys using MRI probes can enable early detection of drug-induced AKI; however, these probes have limitations in monitoring the diverse pathological processes in drug-induced AKI [24,25]. Therefore, noninvasive methods to detect diverse pathological processes in the kidneys are needed. The MRI probe PAH-Gd exhibited programmable responsiveness to H_2_O_2_ and MPO, manifesting as varying degrees of T1WI enhancement *in vitro* and *in vivo*. PAH-Gd could be oxidized by H_2_O_2_ to 5-hydroxytryptamine-DOTA/Gd (H–Gd), which selectively accumulated in PETCs *via* ligand-receptor interactions, moderately increasing the renal T1 WI signal. Subsequently, H–Gd underwent further oxidation by MPO in the presence of H_2_O_2_, leading to the formation of dimers or adducts with adjacent proteins. This process caused a significant elevation in the renal T1 WI signal. Moreover, the blocking experiments with NAC or 4-ABAH not only confirmed the programmable responsiveness of PAH-Gd to H_2_O_2_ and MPO *in vivo* but also elucidated the spatiotemporal detection capabilities of PAH-Gd for renal oxidative stress and inflammation. These results suggested that PHA-Gd could detect the occurrence and development of drug-induced AKI in the early stage.

The gadolinium-based contrast agents (GBCAs) are widely used to enhance MRI in the clinic; however, their potential nephrotoxicity warrants consideration, especially in patients with pre-existing renal impairment. For patients with mild elevations in creatinine and an estimated glomerular filtration rate (eGFR) above 30 mL/min/1.73 m^2^, the use of GBCAs is generally considered acceptable for contrast-enhanced MRI. However, in patients with moderate to severe renal impairment (eGFR <30 mL/min/1.73 m^2^), GBCAs should be avoided. Considering normal renal function in the early stage of drug-induced AKI, PAH-Gd demonstrated renal clearance comparable to Magnevist and exhibited negligible cytotoxicity in the kidneys of normal mice. However, the safety and pharmacokinetics of PAH-Gd need further investigation on different animal models before clinical trials.

In summary, we exploited PAH-Gd for sequential monitoring of renal oxidative stress and inflammation, enabling early detection of the occurrence and progression of DDP-induced AKI. The PAH-Gd not only detected the early stage of DPP-induced AKI with oxidative stress at least 60 h earlier than the standard clinical assays, but also spatiotemporally monitored the progression of renal injury accompanied by an inflammatory response. Under oxidative stress, PAH-Gd is oxidized by H_2_O_2_ to 5-hydroxytryptamine-DOTA/Gd (H–Gd), which promotes the accumulation of Gd^3+^ within proximal tubular epithelial cells (PTECs) *via* ligand-receptor interactions, producing a moderate enhancement of renal T1 WI. Under inflammation, renal T1WI ascended to higher levels through a logical mechanism: 1) the recovery of ligand-receptor interactions by H_2_O_2_, leading to accumulation of Gd^3+^ in PTECs; 2) MPO further catalyzed H–Gd in the presence of H_2_O_2_, forming dimers or generating adducts with nearby proteins, which led to intensive retention and slow tumbling effect of Gd^3+^ in kidneys. PAH-Gd provided a comprehensive spatiotemporal strategy for the early diagnosis of AKI, offering an asset for both drug development and the safe clinical application of potentially nephrotoxic medications, ultimately contributing to improved patient safety and treatment outcomes.

## 4 Experiments

### 4.1 Materials

Serotonin hydrochloride (5-HT, ≥98%), tert-butoxycarbonyl anhydride (≥99%), 4-(Bromomethyl)benzeneboronicacidpinacolester (4-BPE, ≥98%) were purchased from Shanghai Aladdin Biochemical Technology Co., Ltd. 1,4,7,10-Tetraazacyclododecane-1,4,7,10-tetraacetic acid mono-N-hydroxysuccinimide ester (DOTA-NHS) was acquired from Sigma-Aldrich (Shanghai) Trading Co.,Ltd. Gadolinium(III) chloride hexahydrate (99.9%), Sodium bicarbonate (NaHCO_3_, ≥99.8%), Potassium carbonate (K_2_CO_3_, ≥99.9%) were purchased from InnoChem Science & Technology. All the biological agents, including Myeloperoxidase (MPO) and Bovine Serum Albumin (BSA) were obtained from Beyotime Biotechnology.

### 4.2 Synthesis of 5-HT-BOC

Serotonin hydrochloride (5-HT) (2.12 g, 10 mmol) was added in 20 mL of chloroform and stirred continuously under N_2_ conditions. NaHCO_3_ (0.84 g, 10 mmol) was dissolved in 15 mL ultrapure water and added to the above 5-HT suspension liquid. 5 mL of NaCl solution at the concentration of 0.4 g mL^−1^ and 5 mL of chloroform containing tert-butoxycarbonyl anhydride (2.18 g,10 mmol) were dropped into the above reaction mixture successively. The mixture was refluxed at 60 °C for 3 h. After the reaction was completed, the aqueous phase was extracted by chloroform for 2 times. The collected organic phase was washed successively with water and saturated NaCl solution for 2 times, followed by drying with anhydrous sodium sulfate. After stirring for 12 h, the organic phase was collected and removed with a rotary evaporator to obtain 5-HT-BOC (2.53 g, 92%).

### 4.3 Synthesis of PAH-BOC

5-HT-BOC (1.38 g,5 mmol) was added to 15 mL anhydrous DMF and stirred continuously under ice bath conditions. K_2_CO_3_ (1.6585 g, 12 mmol) was added to the above solution and stirred for 10 min in a nitrogen atmosphere. The 4-(Bromomethyl)benzeneboronicacidpinacolester (4-BPE) (1.78 g, 6 mmol) dissolved in 5 mL DMF was dropped into the above reaction mixture and stirred at RT overnight. After completion, the DMF was removed by a reduced-pressure rotary evaporator. The crude product was dissolved in ethyl acetate and washed for 3 times with saturated NH4Cl, and then with saturated NaCl solution. After dried by anhydrous sodium sulfate, the organic phase was removed by vacuum rotary evaporator to obtain PAH-BOC (2.1 g, 85%).

### 4.4 Synthesis of PAH

The obtained PAH-BOC was dissolved in 10 mL DCM, and 2 mL of trifluoroacetic acid (TFA) was added and stirred for 30 min. After completion, the solvent was removed by a vacuum rotary evaporator. The crude product was purified by silica gel column chromatography (dichloromethane/methyl alcohol, v/v, 10:1) to obtain PAH (1.82 g, 87%).

### 4.5 Synthesis of PAH-DOTA

DOTA-NHS (55 mg, 0.11 mmol) and PAH (0.13 mmol) were dissolved in 2 mL of anhydrous DMF. Under ice bath conditions, triethylamine (0.26 mmol) was added to the above reaction solution and stirred continuously for 2 h. Then the reaction solution was further stirred at room temperature for 24 h. The entire reaction was carried out in a nitrogen atmosphere. After completion, 3 mL of water was added to terminate the reaction. Then the crude product was purified with the HPLC method (C_18_ column, 5 to 95% CH_3_CN (0.1% HCOOH) in water (0.1% HCOOH) and freeze-dried to obtain white PAH-DOTA (0.06 g, 76%).

### 4.6 Synthesis of PAH-Gd

PAH-DOTA (1 mmol) was dissolved in 50 mL of sodium acetate buffer (1 M, pH = 6) in a nitrogen atmosphere. Subsequently, gadolinium chloride hexahydrate was dissolved in 10 mL of sodium acetate buffer (1 M, pH = 6), and the above solutions were slowly added. Then, stirred at room temperature for 16 h. Then the crude product was purified with HPLC method (C18 column, 5 to 95% CH_3_CN (0.1% HCOOH) in water (0.1% HCOOH) and freeze-dried to obtain white PAH-DOTA (0.74 g, 80%).

### 4.7 Response of PAH-Gd to H_2_O_2_

PAH-Gd was dissolved in water to prepare a 200 μg/mL solution. Subsequently, different concentrations of H_2_O_2_ were added to this solution. The mixture was incubated at 37 °C for 10 min, followed by HPLC analysis under the following conditions: a mobile phase of 60% methanol and 40% water, a flow rate of 0.8 mL/min, and a detection wavelength of 254 nm. The results were analyzed by comparing peak retention times and the areas under the curve (AUC) to determine H_2_O_2_-induced changes in PAH-Gd‌.

### 4.8 Response of PAH-Gd to MPO + H_2_O_2_

PAH-Gd was dissolved in 0.1 M Tris buffer to prepare a 1 mM solution. Subsequently, H_2_O_2_ and MPO with different concentrations were added, and the mixture was incubated at 37 °C. The UV-VIS absorbance at 350 nm was measured at predetermined time intervals. The PAH-Gd solution containing only H_2_O_2_ or only MPO was prepared for comparison purposes.

### 4.9 Cytotoxicity

HK-2 cells were purchased from Srevicebio and cultured in MEM medium supplemented with 10% fetal bovine serum (FBS) and 1% penicillin-streptomycin (P/S) at 37 °C in a constant-temperature incubator under a 5% carbon dioxide atmosphere. HK-2 cells were seeded at a density of 3000 cells per well into 96-well plates and cultured overnight. Different concentrations of PAH-Gd were then added, and the cells were incubated for another 24 h. Subsequently, 20 μL of MTT (5 mg/mL) was added to each well, and the plates were incubated at 37 °C for 4 h. All the medium was carefully removed, and 150 μL of DMSO was added to each well. After gentle shaking, the absorbance at 570 nm was measured using a microplate reader.

### 4.10 DDP-induced AKI mouse models

Mice were pre-deprived of water for 8 h and then intraperitoneally injected with a cisplatin solution (20 mg kg-1). AKI models were established at predefined time points following the injection.

### 4.11 *In vitro* MRI measurement

MRI studies were performed on a 5.0 T MR scanner, and the longitudinal relaxation time (T1) was measured using a parameter of echo time (TE) = 650 ms, eight repetition times (TR) = 12 ms. The phantom samples with different concentrations of PAH-Gd, PAH-Gd + H_2_O_2_, PAH-Gd + H_2_O_2_ + MPO, PAH-Gd + BSA, PAH-Gd + H_2_O_2_ + BSA, and PAH-Gd + H_2_O_2_ + MPO + BSA were prepared and studied by T1 MRI. The final concentration of H_2_O_2_ was 1 mM, and the final concentration of MPO was 200 mU mL-1.

### 4.12 *In vivo* performance of PAH-Gd

All animal experimental procedures were approved by the Experimental Animal Welfare Ethics Committee, Zhongnan Hospital of Wuhan University (ZN2021232). All animal experiments were conducted using a 5.0 T MR scanner to acquire T1 MRI using the parameters of echo time (TE) = 900 ms, eight repetition times (TR) = 11.2 ms. The mice were anesthetized with isoflurane and acquired T1 MRI as blank controls. Then, Magnevist, H–Gd, or PHA-Gd (gadolinium concentration: 50 μM/kg) was injected via the tail vein, and T1 MRI was acquired at different time points after *i.v.* injection.

To measure the T1 WI signal of kidneys (Sk), a region of interest (ROI) was manually traced, encompassing the renal parenchyma or pelvis. To measure the T1 WI signal of background (without tissues) (Sa), a ROI was placed in the field of view without any tissue (air). We analyzed more than 4 coronal slices per mouse (3 mouse per group) across the entire kidneys in this fashion.

SNR (signal-to-noise ratio, %) = 100 × Sk/Sa

△SNR = SNR_post_-SNR_pre_

SNR_post_: the SNR calculated from mouse after treated with pobe.

SNR_pre_: the SNR calculated from mouse before treated with pobe.

### 4.13 The immunofluorescence staining of the kidneys

Fluorescence staining for H_2_O_2_ and MPO in renal tissue was performed accordance with standardized commercial staining kits.

To observe renal H_2_O_2_, the 10 μm unfixed frozen sections, prepared from fresh kidneys, were stained with BBoxiProbe O32 (10 μL) at 37 °C for 60 min in the dark. After washed three times with PBS, the renal sections were incubated with 4′,6-diamidino-2-phenylindole (DAPI) for 15 min, then washed three times with PBS. At last, the renal sections were observed with CLSM.

The Tyramide Signal Amplification (TSA) technology was applied to observe renal MPO. Generally, after deparaffinization and retrieval (citrate or Tris-EDTA buffer, pH 6.0/8.0/9.0, 25 min), endogenous peroxidase in sections was blocked with 3% H_2_O_2_ in methanol. The sections were then blocked with 3% BSA and incubated with a rabbit anti-MPO primary antibody (Servicebio, GB15006, 1:3000) overnight at 4 °C, followed by a horseradish peroxidase-conjugated goat anti-rabbit polymer secondary antibody (50 min, RT) and iF647-Tyramide (10 min, RT). Then, the cellular nuclei were stained with DAPI, and autofluorescence was quenched before mounting. At last, the renal sections were observed with CLSM.

### 4.14 Biocompatibility of PAH-Gd

PAH-Gd (50 μM/kg) or PBS was intravenously injected into the tail vein of mice. Three days post i.v. injection, the mice were sacrificed, and the pathological damage of major organs, including the heart, liver, spleen, lungs, and kidneys was observed by HE staining.

### 4.15 Blood biochemical analysis

The mice were anesthetized with ‌isoflurane‌, and blood‌ was collected from eye socket. The blood samples were centrifuged at ‌3500 rpm/min for 15 min‌ to obtain serum. The ‌ serum creatinine (sCr), ood urea nitrogen (BUN), and uric acid‌ in serum were measured using commercial assay kits according to the manufacturer's protocol.

### 4.16 Statistical analysis

All the statistical analyses were performed using GraphPad Prism 7.0. Statistical analyses were performed using one-way analysis of variance (ANOVA) followed by Tukey's honestly significant difference (HSD) test for multiple comparisons. The statistical data were presented as Mean ± SD. A statistical significance of P < 0.05 was selected, and ∗ indicated P < 0.05, ∗∗ indicated P < 0.01, and ∗∗∗ indicated P < 0.001, respectively.

## Data availability statement

The data that support the findings of this study are available from the corresponding author upon reasonable request.

## CRediT authorship contribution statement

**Li He:** Data curation, Formal analysis, Methodology. **Jia-Mi Li:** Investigation, Methodology, Project administration, Resources, Validation. **Meng-Ting Li:** Visualization. **Cai-Ju Zhang:** Investigation. **Yu-Fan Lv:** Formal analysis. **Jiao-Jiao Ma:** Methodology. **Mao-Lin Zou:** Software. **Bo Wu:** Resources. **Shi-Wen Huang:** Validation. **Gang Liu:** Supervision. **Yong-Chang Wei:** Supervision, Validation. **Dan Xu:** Project administration, Supervision, Visualization. **Kai Deng:** Conceptualization, Funding acquisition, Project administration, Writing – original draft, Writing – review & editing.

## Declaration of competing interest

The authors declare that they have no competing interests.
