# Supplementary material for: An H2O2 and MPO programmable responsive MRI probe for early detection of drug-induced acute kidney injury via spatiotemporal monitoring of renal oxidative stress and inflammation
Source: Redox Biol. 2026 Jun 25;95:104278. doi: 10.1016/j.redox.2026.104278 (PMC13324336; doi:10.1016/j.redox.2026.104278)
Supplement: Multimedia component 1 [file mmc1.docx]

An H_2_O_2_ and MPO programmable responsive MRI probe for early detection of drug-induced acute kidney injury *via* spatiotemporal monitoring of renal oxidative stress and inflammation

Li He ^a,c,1^, Jia-Mi Li ^b,1^, Meng-Ting Li ^a^, Cai-Ju Zhang ^a,g^, Yu-Fan Lv ^c^, Jiao-Jiao Ma ^a^ Mao-Lin Zou ^a^, Bo Wu ^a^, Shi-Wen Huang ^f^, Gang Liu ^e^, Yong-Chang Wei ^c,*^, Dan Xu ^d,**^, Kai Deng ^a,***^

^a^ Department of Radiology, Zhongnan Hospital of Wuhan University, Wuhan 430071, People’s Republic of China

^b^ Department of Radiology, Renmin Hospital of Wuhan University, Wuhan, 430060, People’s Republic of China

^c^ Department of Radiation and Medical Oncology, Zhongnan Hospital of Wuhan University, Wuhan 430071, People’s Republic of China

^d^ Department of Nuclear Medicine, Zhongnan Hospital of Wuhan University, Wuhan 430071, People’s Republic of China

^e^ State Key Laboratory of Molecular Vaccinology and Molecular Diagnostics, State Key Laboratory of Vaccines for Infectious Diseases, Center for Molecular Imaging and Translational Medicine, Xiangan Biomedicine Laboratory, School of Public Health, Xiamen University, Xiamen 361102, People’s Republic of China

^f^ Key Laboratory of Biomedical Polymers of Ministry of Education, Department of Chemistry, Wuhan University, Wuhan 430072, People’s Republic of China

^g^ Department of Radiology, Hainan Affiliated Hospital of Hainan Medical University, Hainan 570311, People’s Republic of China

* Corresponding author. Department of Radiation and Medical Oncology, Zhongnan Hospital of Wuhan University, Wuhan 430071, People’s Republic of China

** Corresponding author. Department of Nuclear Medicine, Zhongnan Hospital of Wuhan University, Wuhan University, Wuhan 430071, People’s Republic of China

*** Corresponding author. Department of Radiology, Zhongnan Hospital of Wuhan University, Wuhan 430071, People’s Republic of China

*E-mail addresses:* [weiyongchang@whu.edu.cn](mailto:weiyongchang@whu.edu.cn) (Y.-C. Wei), [xudan942004@whu.edu.cn](mailto:xudan942004@whu.edu.cn) (D. Xu), deng-k@whu.edu.cn (K. Deng)

^1^ These authors contributed equally to this work.


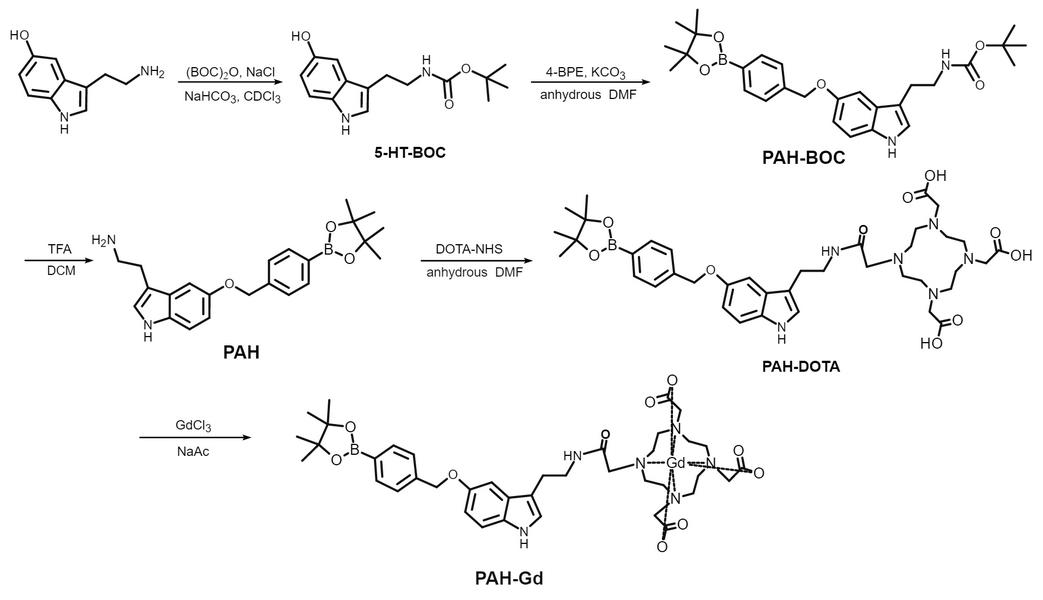
**Fig. S1.** The synthetic route of PAH-Gd.


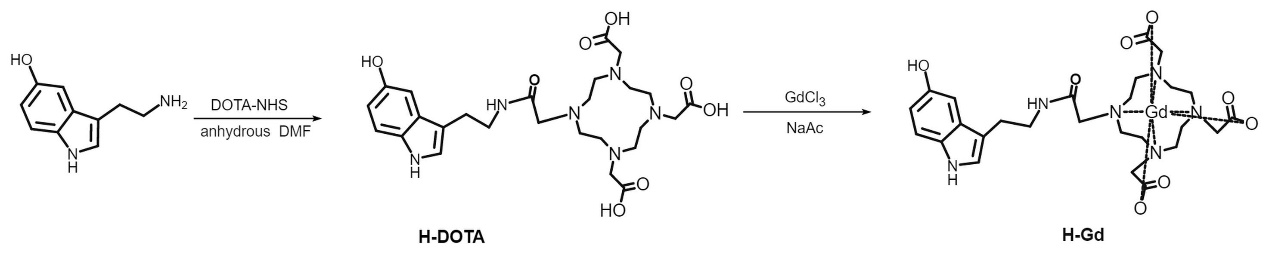
**Fig. S2.** The synthetic route of H-Gd.


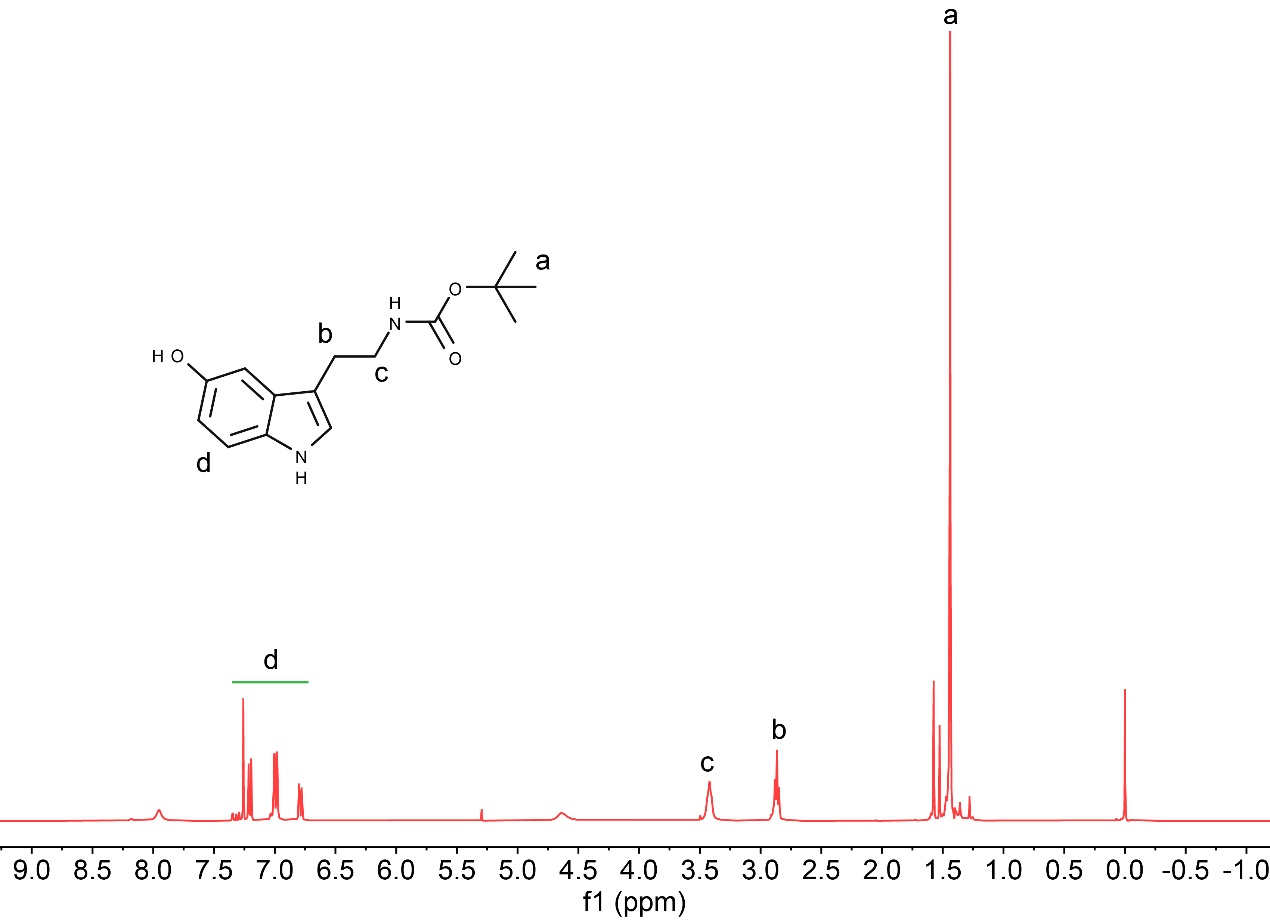
**Fig. S3.** ^1^H NMR of 5-HT-BOC in DMSO-*d_6_*.


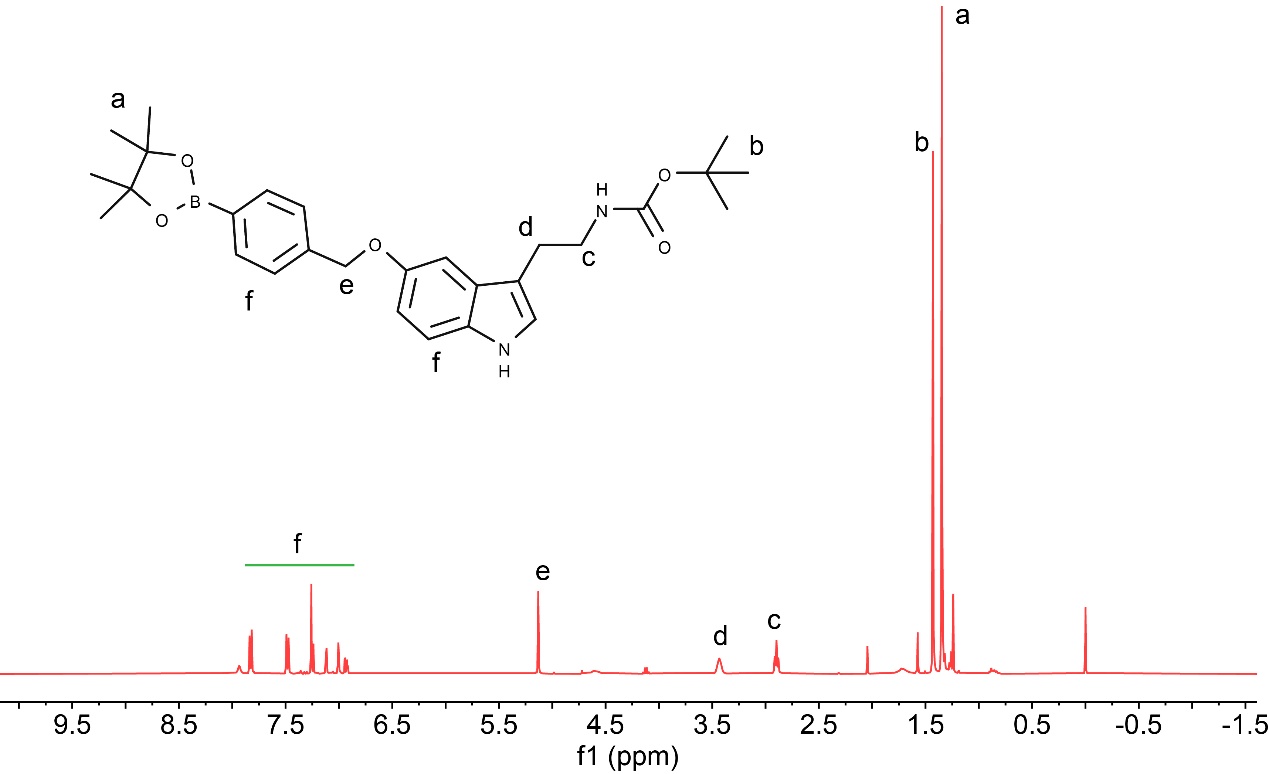
**Fig. S4.** ^1^H NMR of PAH-BOC in DMSO-*d_6_*.


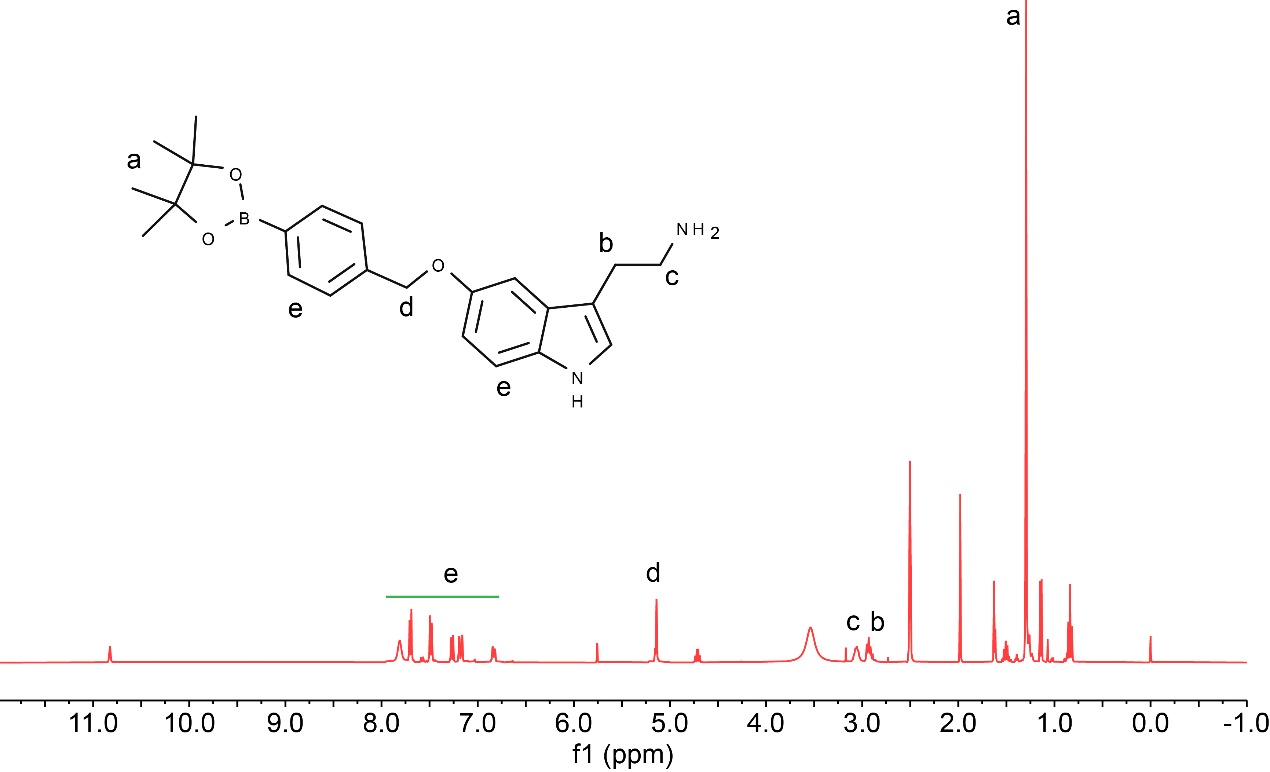
**Fig. S5.** ^1^H NMR of PAH in DMSO-*d_6_*.


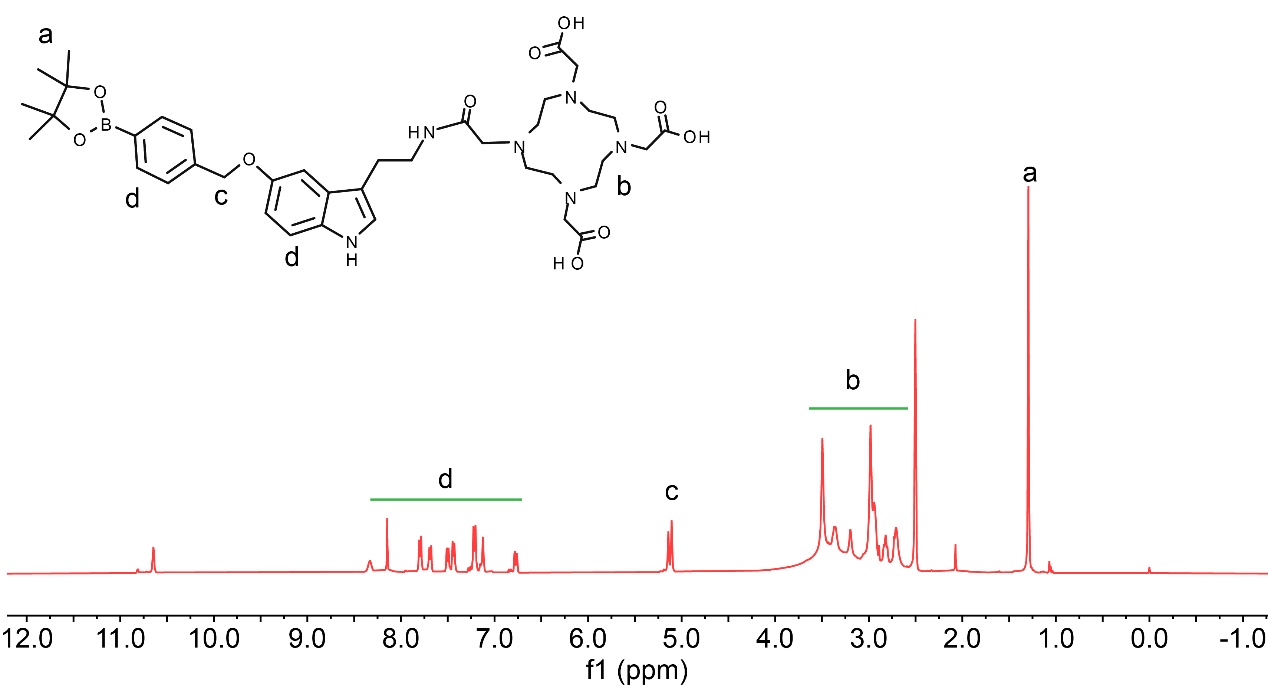
**Fig. S6.** ^1^H NMR of PAH-DOTA in DMSO-*d_6_*.


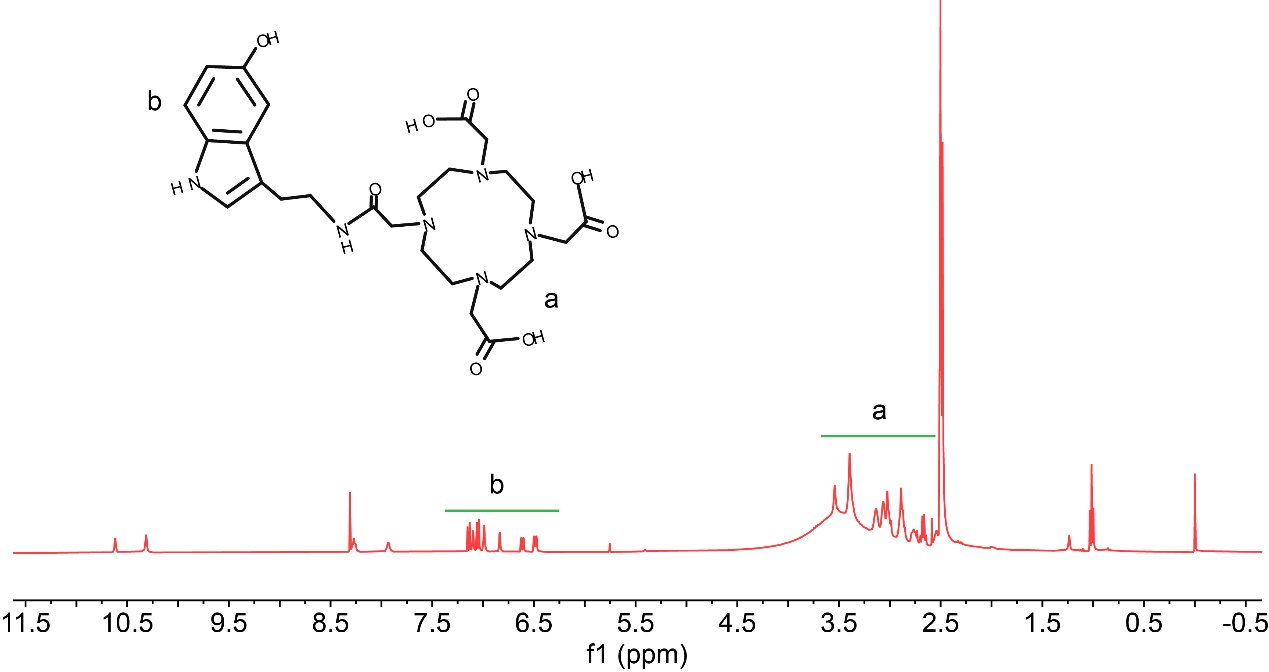
**Fig. S7.** ^1^H NMR of H-DOTA in DMSO-*d_6_*.


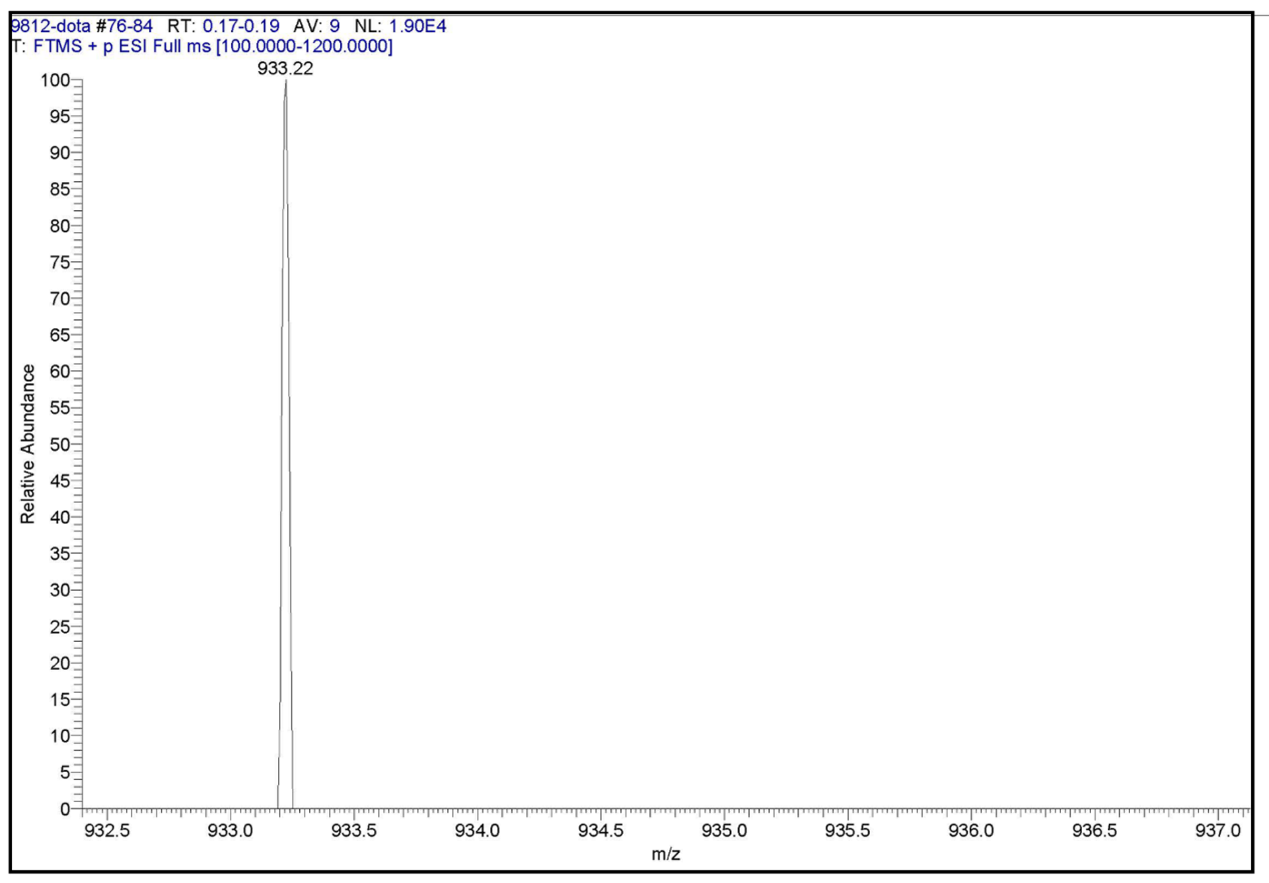

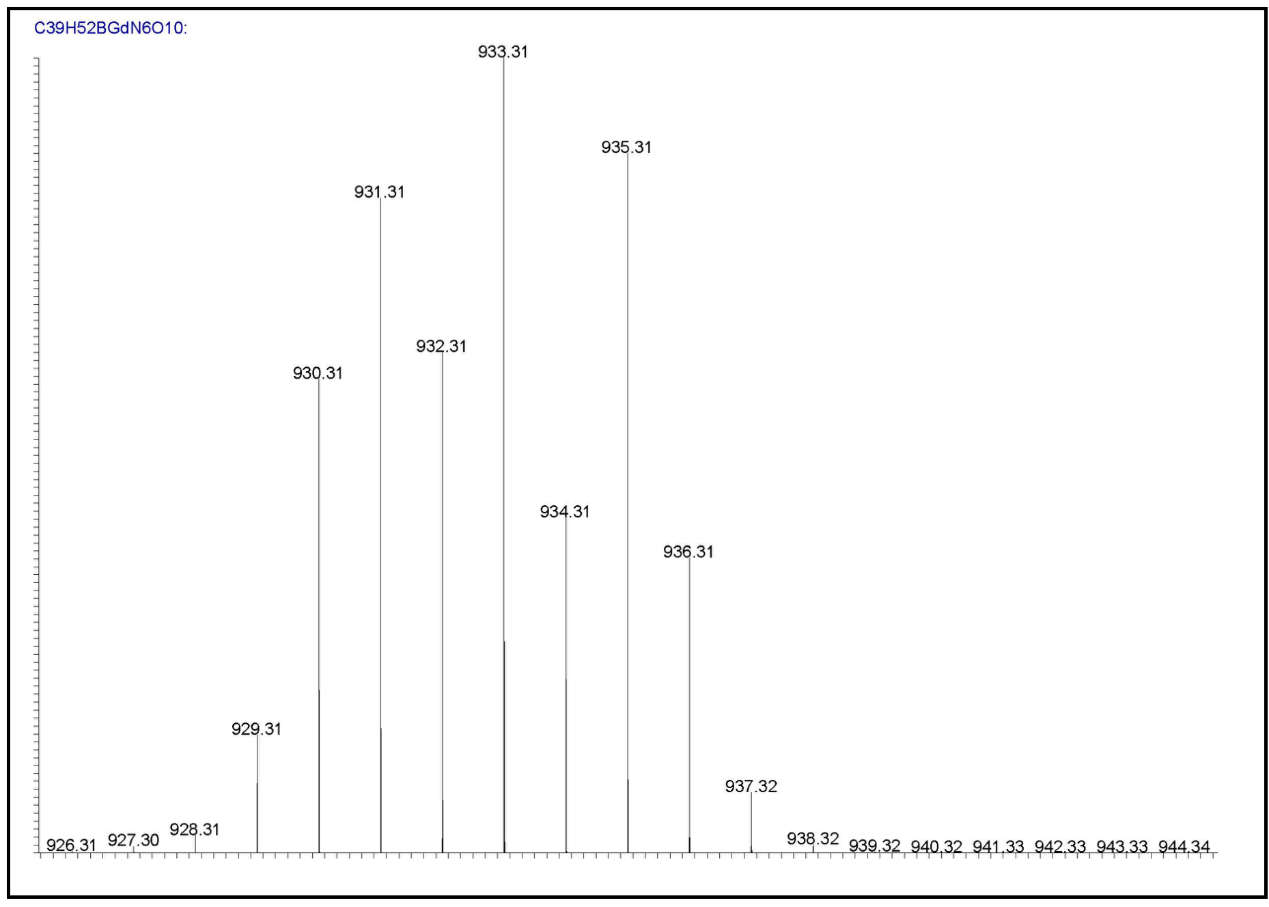
**Fig. S8.** HR-MS of PAH-Gd (top) and theoretical pattern (bottom).

**Fig. S9**. HR-MS of H-Gd (top) and theoretical pattern (bottom).

**Fig. S10.** HPLC analysis of the PAH-Gd treated with H_2_O_2_ (1 mM) for different times.


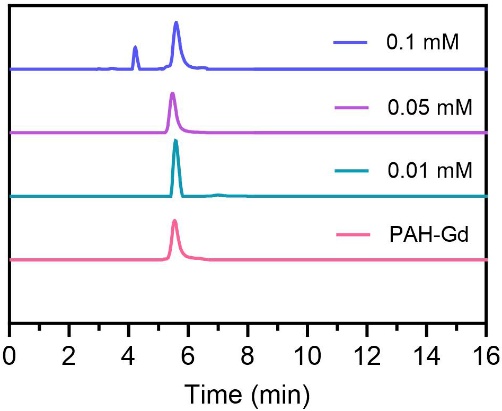


**Fig. S11.** The HPLC analysis of the PAH-Gd treated with different concentration of H_2_O_2_ (0.01, 0.05, 0.1 mM) for 10 min.

**Fig. S12.** The UV–Vis absorbance at 350 nm for PAH-Gd with different concentrations of H_2_O_2_ in the presence of MPO (0.1M Tris buffer, pH = 7.4, 37 °C). Data are means ± SD (n = 3).


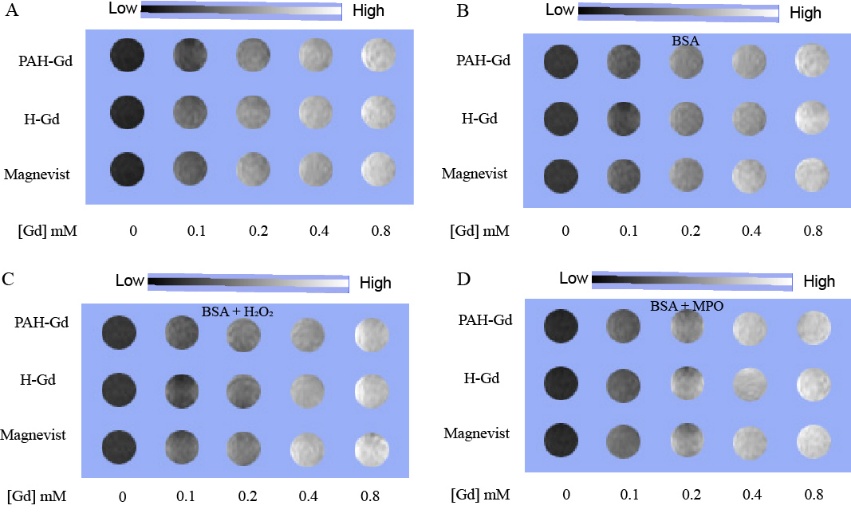


**Fig. S13.** (**A**) The T1 WI phantom images of PAH-Gd, H-Gd and magnevist. The T1 WI phantom images of PAH-Gd, H-Gd and magnevist in the presence of BSA, following MPO (**B**), H_2_O_2_ (**C**) and MPO(**D**) stimulation.


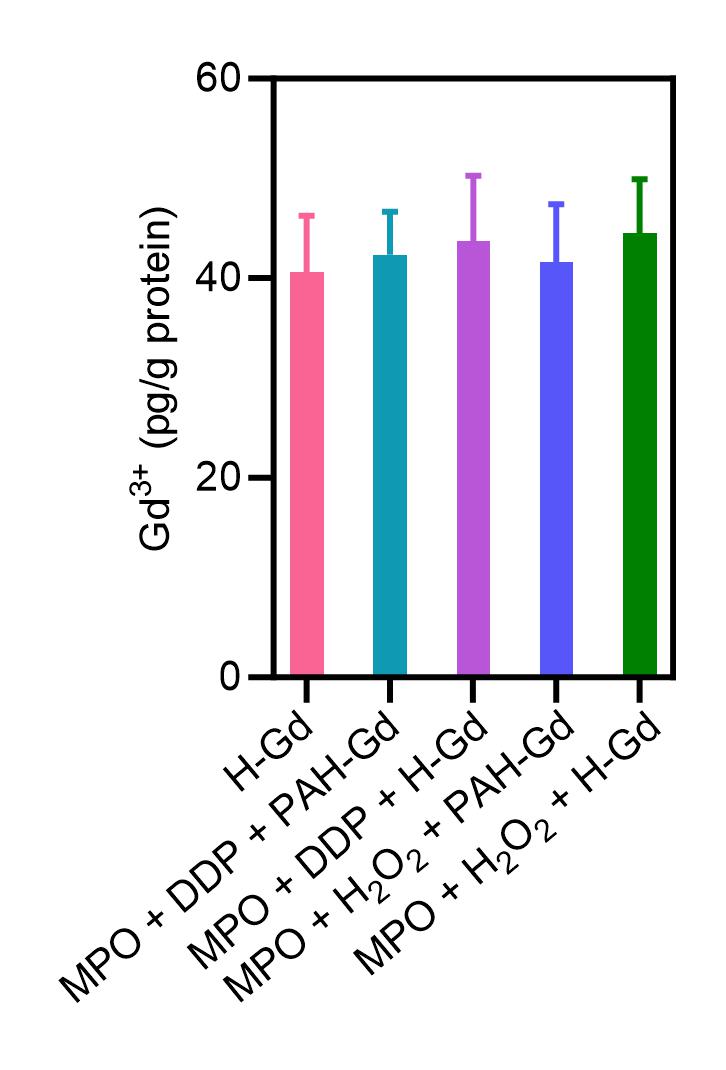


**Fig. S14.** The ICP analysis of cellular concentration of Gd^3+^ after different treatments. Data are means ± SD (n = 3).


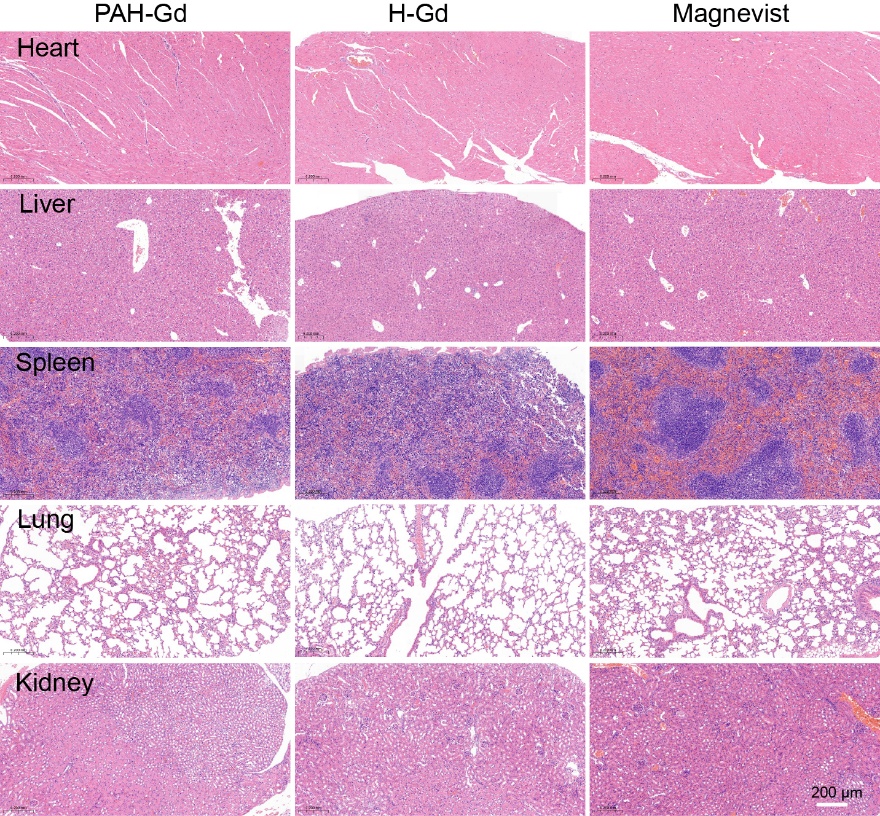


**Fig. S15.** HE staining of major organs after mice treated with PAH-Gd, H-Gd, and Magnevist for 3 days.
